# Land use and land cover change detection and spatial distribution on the Tibetan Plateau

**DOI:** 10.1038/s41598-021-87215-w

**Published:** 2021-04-06

**Authors:** Shuang Hao, Fengshun Zhu, Yuhuan Cui

**Affiliations:** 1 School of Natural Science, Anhui Agricultural University, Hefei, 20036 China; 2 College of Geodesy and Geomatics, Shandong University of Science and Technology, Qingdao, 266590 China

**Keywords:** Environmental sciences, Solid Earth sciences

## Abstract

Regarded as the third pole of the Earth, the Tibetan Plateau (TP) is a region with complex terrain. Vegetation is widely distributed in the southeastern part of the plateau. However, the land use and land cover changes (LULCC) on the TP have not been sufficiently studied. In this study, we propose a method of studying the dynamic changes in the land cover on the TP. Landsat OLI images (2013 and 2015) were selected to extract the LULCC information of Nyingchi County, the DEM was used to extract objects’ land curved surface area and analyze their three-dimensional dynamic change information, which realized a four-dimensional monitoring of the forestry information on time and spatial level. The results showed that the forest area in 2015 decreased by 7.25%, of which the coniferous forest areas decreased by 25.14%, broad-leaved forest areas increased by 12.65%, and shrubbery areas increased by 14.62%. Compared with traditional LULCC detection methods, the change detection is no longer focused on the two-dimensional space, which helps determine the three-dimensional land use and land cover changes and their distribution. Thus, dynamic spatial changes can be observed. This study provides scientific support for the vegetation restoration and natural resource management on the TP.

## Introduction

With an average altitude for over 4000 m, the Tibetan Plateau (TP) is called the water tower of Asia. It is the cradle of the Yangtze, Yellow, Yarlung Zangbo, Ganges and Indus rivers, and it provides water resources for more than 2 billion people^1–3^. The total area of the TP is about 2.5 million km^2^, accounting for a quarter of China’s total land area. Due to the special terrain features and the unique thermal and dynamic processes, the TP not only affects the climate of Eastern Asia, but it also has a significant influence on the global climate. A unique ecosystem has formed on the TP due to its unique geographical and climatic characteristics. The ecosystem of the TP is fragile and vulnerable, and it is exceedingly sensitive and vulnerable to natural changes and anthropogenic disturbances^4–7^. Although the ecosystem of most of the TP is not ideal for vegetation growth, vegetation is distributed across most parts of the TP. Grasslands are extensively distributed over the TP, and forestlands is widely distributed in the southeastern part of the plateau. According to the previous studies, some of the grasslands and forest land are undergoing degradation due to the climatic and non-climatic drivers^8–10^. The TP is sensitive to climate change, and its response to climate change is about 4–8 years ahead of the rest of the Earth, which makes it an important indicator of global change^11^. Land use and land cover changes (LULCC) reflect the anthropogenic forcing behaviors on the Earth’s surface. They are the main contributor to global climate change and are viewed as one of the influential environmental issues for worldwide consideration^12–16^. Former studies have demonstrated that the TP affects the global climate, and it is a crucial region for the Earth ecosystem^17–19^. The vegetation cover and its spatial distribution have significant effects on the framework and function of the ecosystem. As an important component of the ecosystem, vegetation plays an important role in climate change^20,21^, and it is also a dominant factor in the maintaining the functions of the terrestrial ecosystem^22,23^.

On the landscape scale or even smaller scales, the land cover types are predominantly affected by non-terrain environmental factors. But for the study of forest coverage on regional scale or even larger scale, the terrain factors cannot be ignored, for they are one of the most important environmental factors that impacts the forestry distribution by controlling the redistribution of the radiation, heat, moisture and soil nutrients. The distribution and ecology of alpine ecosystems are dominated by cold climatic conditions, but the status of the ecosystem varies with the length of the growing season^24^. Alpine ecosystems are globally important due to both their biodiversity and the ecosystem they provide for humans^25,26^. Vegetation is an important component of the alpine ecosystem. It is also an important indicator of the regional ecological environment. The structural functions and ecological features of the alpine vegetation reflect the ecological environment’s conditions. Furthermore, the vegetation types are the foundation for the study of vegetation coverage and dynamic changes. Therefore, studying the alpine vegetation and its dynamic changes is important to understanding the alpine ecological environment and climate change.

Studying the relationship between the vegetation distribution and the terrain condition could provide guidance for the exploration of the heterogeneity of the forest land and spatial distribution of the forestry resources. A digital elevation model (DEM) is a quantitative representation of the Earth’s surface and the spatial information data, so it has become a vital source of information about the elevation, slope, and terrain in scientific investigations^27,28^. The remote sensing technique and DEM data have been widely used in land feature investigations, and these techniques and data can help improve the analytical ability of geoscience research^29^. For the past few decades, the TP has suffered from climate change and increasing temperatures, which have been aggravated in recent years. However, only a few studies have been conducted on the LULCCs on the TP, and precise and adequate information about land use change detection is extremely important for preferable management^30^. Therefore, in this study, a basic method for land cover change detection was applied to extract the land cover change information from 2013 to 2015, and Landsat OLI images were used as the main satellite data source. Previous studies rarely consider the impact of the topography on the land use type changes monitoring, the digital elevation model (DEM) is rarely used to study and extract the objects’ land curved surface area. Therefore, the three-dimensional spatial information of the forestry resources changes has not been finely deep mined. Thus, in this study, the DEM data were used to analyze the spatial distribution of and dynamic changes in the land cover on the TP. The objective of this study was to accurately describe the land cover changes in the alpine vegetation on south-eastern TP from 2013 to 2015. As the terrain factor is considered in this study, the land use type and land cover change information are not confined in the two-dimensional plane level, which realized a four-dimensional monitoring of the mountainous forestry information, on the time and spatial level. With combination use of DEM and Landsat OLI images, the three-dimensional terrain visualization is realized, a real three-dimensional forestry land cover distribution and change monitoring were generated. In this study, the mapping relationship between the forest vegetation and the image features was established, which can improve the accuracy of information’s spatial expression, it also can help the forest resource monitoring in the region of complex terrain. The results of this study are significant for natural resource conservation and vegetation resource management in alpine areas.

## Results

**Figure 1:**
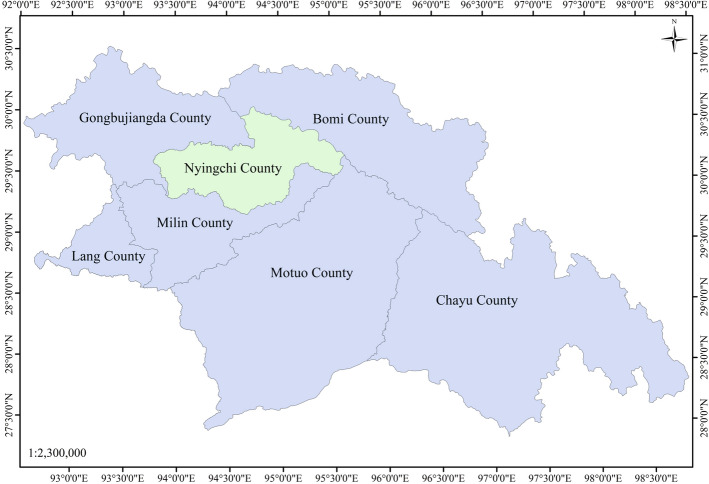
The location of Nyingchi County. The map is generated using ArcGIS 10.3 (http://www.esri.com/software/arcgis/arcgis-for-desktop).

Nyingchi County (93°27′–95°17′ E, 29°21′–30°15′ N) is located in the southeastern part of the Tibetan Autonomous Region of China (Fig. 1). The Yarlung Zangbo River, which is the largest river in China and one of the highest rivers in the world, flows through Nyingchi County. The study area is in a vegetation covered high mountain area with large topographic relief. The average altitude of the study area is 3000 m. The highest summit of the Nyingchi County is Gyala Peri Mountain, which is about 7294 m above sea level. It is the 85^th^ highest mountain in the world. The southwestern warm and humid air makes Nyingchi County a fertile area with abundant species.

### Land use and land cover change detection

**Figure 2:**
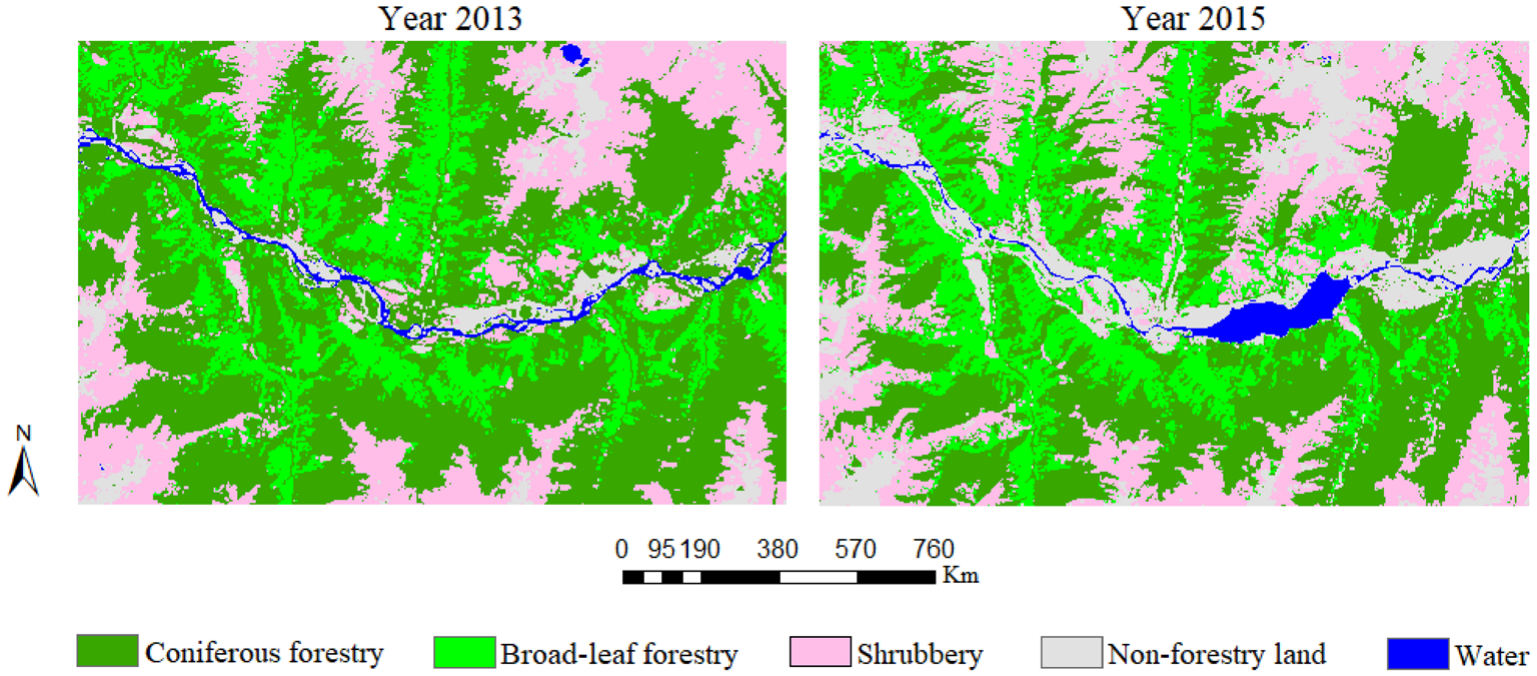
The maximum likelihood classification results of 2013 and 2015. The figure is generated using ArcGIS 10.3 (http://www.esri.com/software/arcgis/arcgis-for-desktop).

Based on the forestry inventory data and the field sampling data, the land cover types in the study area were divided into five types: coniferous forest (CF), broad-leaf forest (BF), shrubbery (SH), non-forestry land (NF), and water (WA). Using the ENVI 5.3 platform, the maximum likelihood classification method was using for remote sensing image classification. The classification results are shown in the following Fig. 2.

Maximum likelihood classification method was applied to extract the land cover information of the Landsat OLI images in 2013 and 2015, the forestry resource inventory data and sampling data were used as classification and validation samples, the confusion matrix was used to verify the classification accuracies, according to the verified results, the classification accuracies were 85.79% (2013) and 87.17% (2015). After obtaining the land cover classification results for the study area, the change detection statistics were calculated to obtain the land use transition matrix of the study area. Which is shown in Table 3. In Table 1, the diagonal elements are the plane projection areas of the land use types that did not change, i.e., land use unchanged (LUU), the off-diagonal elements are the plane projection areas of the land use types that did change, i.e., land use changed (LUC).

**Table 1:** The projection areas of the curved surface in the changed area.

Areas (m^2^)	13 CF	13 BF	13 SH	13 NF	13 WA
15 CF	138,000,000	15,351,300	4,710,600	288,000	14,400
15 BF	29,766,600	62,545,500	3,151,800	257,400	3600
15 SH	32,712,300	7,131,600	71,939,700	1,804,500	18,000
15 NF	10,612,800	1,544,400	20,230,200	23,378,400	3,083,400
15 WA	1,377,900	26,100	694,800	2,994,300	2,898,900

### Dynamic land cover changes on the curved surfaces

Based on the curved land surface areas of the 20 LUCs and 5 LUUs, a new land use transition matrix was obtained. The statistical land use change data for 2013 and 2015 is shown in Table 2, and the statistical land use area data for 2013 and 2015 is shown in Table 3.

**Table 2:** Statistical dynamic land cover changes statistical data for 2013 and 2015.

Land cover type	Area in 2013 (m^2^)	Area in 2015 (m^2^)	Area by percentage in 2013 (%)	Area by percentage in 2015 (%)	Area dynamic change (m^2^)	Areas dynamic change percentage (%)
CF	268,415,185.9	200,944,061.7	50.04	37.46	− 67,471,124.22	− 25.14
BF	106,469,628.2	119,935,143.6	19.85	22.36	13,465,515.42	12.65
SH	122,608,249.1	140,530,146.3	22.86	26.20	17,921,897.19	14.62
NF	32,581,499.13	66,696,334.92	6.07	12.43	34,114,835.79	104.71
WA	6,293,179.35	8,262,055.17	1.17	1.54	1,968,875.82	31.29
Total area of forestland	497,493,063.2	461,409,351.6	92.75	86.02	− 36,083,711.61	− 7.25
Total area	536,367,741.7	536,367,741.7	100.00	100.00	0	0.00

**Table 3:** Land cover area transfer data between 2013 and 2015.

Area (m^2^)	2013 CF	2013 BF	2013 SH	2013 NF	2013 WA	Decrease	Transfer-out rate
2015 CF	175,687,800	18,845,255.88	6,038,518.14	357,955.2	14,532.48	92,727,385.92	34.55%
2015 BF	38,803,739.76	76,824,637.65	3,985,766.28	317,399.94	3600	29,644,990.56	27.84%
2015 SH	41,240,396.61	9,119,890.08	88,075,774.71	2,075,535.9	18,549	34,532,474.4	28.16%
2015 NF	11,264,425.92	1,651,426.92	23,796,784.26	26,716,835.52	3,266,862.3	5,864,663.61	18.00%
2015 WA	1,418,823.63	28,417.68	711,405.72	3,113,772.57	2,989,635.57	3,303,543.78	52.49%
Increase	25,256,261.7	43,110,505.98	52,454,371.59	39,979,499.4	5,272,419.6		
Transfer-in rate	87.43%	64.06%	62.67%	40.06%	36.19%		

According to the classification results, over 80% of the land in the study area is covered by forests. Based on the classification results for 2015, 37.46% of the study area’s land was covered by CF, which is the most widely distributed land use cover type. The SH accounted for 26.20% of the land area, and 22.36% of the land in the study area was BF. The rest of the land area was NF, accounting for about 12.53%, and only 1.54% of the land in the study area was WA. Compared with the classification results for 2013, the NF had the highest net increment of all the land cover types, the NF area increased by 34,114,835.79 m^2^. The net increments of the SH, BF, and WA were 17,921,897.19 m^2^, 13,465,515.42 m^2^, and 1,968,875.82 m^2^, respectively. However, the CF area decreased by about 67,471,124.22 m^2^.

The statistical data show that the total forestry area decreased by 7.25%, of which the SH area increased by 14.62% and the CF area decreased by 25.14%. According to the comparison, the NF had a significant increment. Its area increased by 104.71% in 2015, while the WA increased by 31.29%.

Table 3 shows the land cover area transfer data for the study area for 2013 and 2015. Based on the data presented in Table 3, the CF area decreased by 92,727,386 m^2^, which is the largest change of all the land cover types in the study area. Most of the CF area in 2013 was transformed into SH and BF, the transformed areas were 41,240,396.61 m^2^ and 38,803,739.76 m^2^, respectively. The decrease in the SH area was 34,532,474.4 m^2^, and most of the SH was transformed into NF. The BF area decreased by 29,644,990.56 m^2^, and most of the BF area in 2013 was transformed into CF, of which the transformed area was 18,845,255.88 m^2^.

### Spatial dynamic change of the land cover

**Figure 3:**
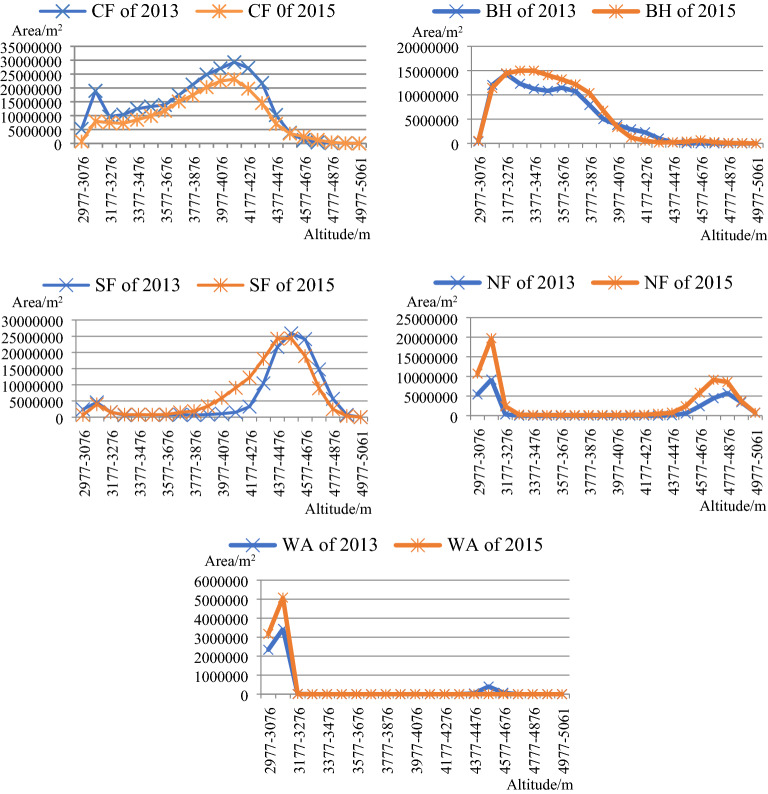
Distribution of the different land cover types in the 21 altitude gradients in 2013 and 2015. The figure is generated using the Microsoft Office 2016 (http://www.office.com).

The highest and lowest altitudes in the study area are 5061 m and 2977 m. Based on altitude, the study area was divided into 21 altitude gradients, with an interval size of 100 m. The curved land surface areas of the CF, BF, SH, NF, and WA in the different altitude gradients were calculated for both 2013 and 2015. The line graphs created from the calculation results are shown in Fig. 3.

According to Fig. 3, the CF area significantly decreases in the 3077–3176 m and 4077–4176 m altitude gradients. Within the 3577–4176 m altitude gradients, the CF area’s reduction level increased as the altitude increased. Within the 4177–4576 m altitude gradient, the CF area’s reduction level decreased as the altitude increased. Most of the increases in the CF area occurred in the 4577–4876 m altitude gradients. In the 3177–3976 m altitude gradients, the BF area increased, but the increase was only slightly correlated with the altitude. However, as the altitude increased to 3977–4376 m, the BF area decreased. The SH area increased in the 3777–4476 m altitude gradient, whereas in the 4477–4876 m altitude gradient, it decreased. The SH was mainly distributed at lower altitudes; however, very little SH occurred in the 3277–3776 m altitude gradient. Most of the increases in the NF area occurred in the 2977–3276 m and 4477–4876 m altitude gradient; very little NF occurred in the 3277–4376 m altitude gradient. The WA area increased in the 2977–3176 m altitude gradient, due to the interception of the dam and water storage project, there is a significant increase in the area of upstream water bodies. Most of the water body increase areas were transferred from the CA and SH, not too much BF can be found transferred into WA in this altitude gradient; very little WA occurred in the 3177–5061 m altitude gradient. Overall, the areas change of WA is closely related to human activity at the lower altitude region, which include socio-economic construction and infrastructure construction.

Based on LUC and LUU DEM data for the CF, BF, and SH, the sum of the relevant curved land surface areas in the 21 altitude gradients were calculated. Based on the line charts, their distributions were analyzed, and the cloud point data were used to create a three-dimensional topographic map. The three-dimensional topographic map of the CF, BF, and SH in both the LUC and LUU areas overlap with the three-dimensional topographic map of the study area. Therefore, the dynamic changes in the forest land on three-dimensional map were obtained. Based on this three-dimensional map, the spatial dynamic changes in the forest land cover were analyzed.

**Figure 4:**
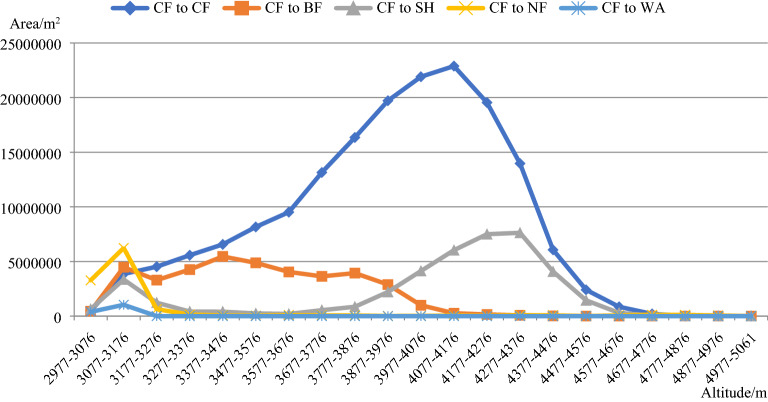
Distribution of the CF changes and the unchanged areas in the 21 altitude gradients between 2013 and 2015. The figure is generated using the Microsoft Office 2016 (http://www.office.com).

According to Fig. 4, in the 2977–4176 m altitude gradient, as the altitude level increased, the unchanged CF area decreased and very few unchanged CF areas occurred in the 4777–5061 m altitude gradient. The water vapor carried by the monsoon crosses the mountain and heats up on the leeward slope, there is more precipitation in the high slopes than in the valleys. Therefore, the CF conservation areas are widely distributed at high altitudes. In the 3077–3976 m altitude gradient, the CF transformed into BF occurred in the 3077–3976 m altitude gradient. The CF mainly transformed into SH in the 3077–3176 m and 3877–4576 m altitude gradient; the CF mainly transformed into NF in the 2977–3176 m altitude gradient; and the CF only transformed into WA in the 2977–3176 m altitude gradient.

**Figure 5:**
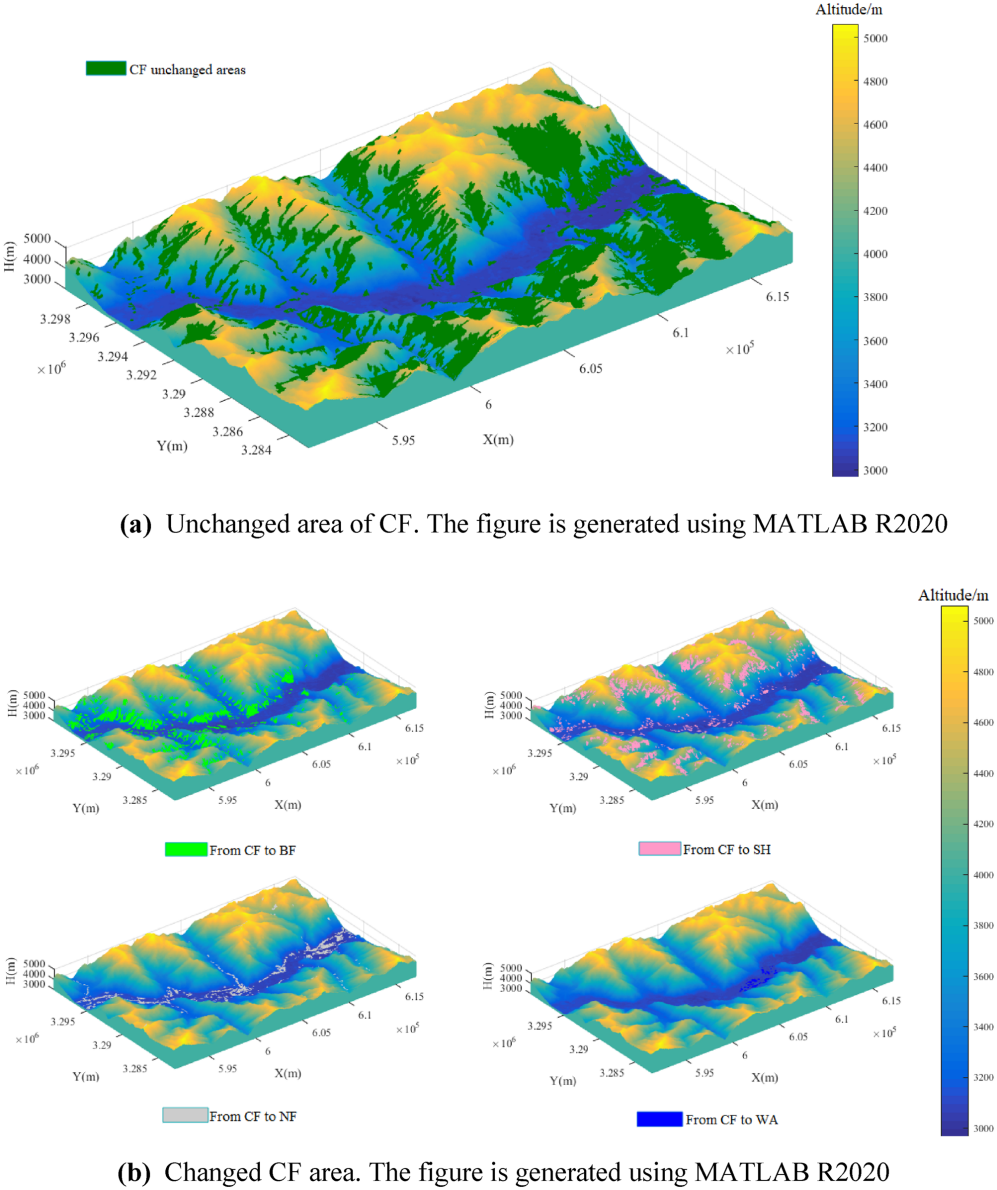
**(a)** Unchanged area of CF. The figure is generated using MATLAB R2020 (http://www.mathworks.com). **(b)** Changed CF area. The figure is generated using MATLAB R2020 (http://www.mathworks.com).

Figure 5a shows that the unchanged CF area was mainly distributed on the northern aspect of the mountains, and very little of the unchanged CF area was distributed in the southern and eastern aspect of the mountains.

Figure 5b shows that the CF mainly transformed into BF in the western part of the study area, and its distribution has no direct relationship with mountains. Thus, the CF transformed into BF for different directions in the mountains, and it was mainly distributed in the 3200–3800 m altitude gradient. In the northern part of the study area, the CF was transformed into SH, mainly on the eastern and southern aspects of the mountain. Along the river, the CF transformed into NF. Very little CF transformed into WA, and this transformation was mainly caused by water storage projects.

**Figure 6:**
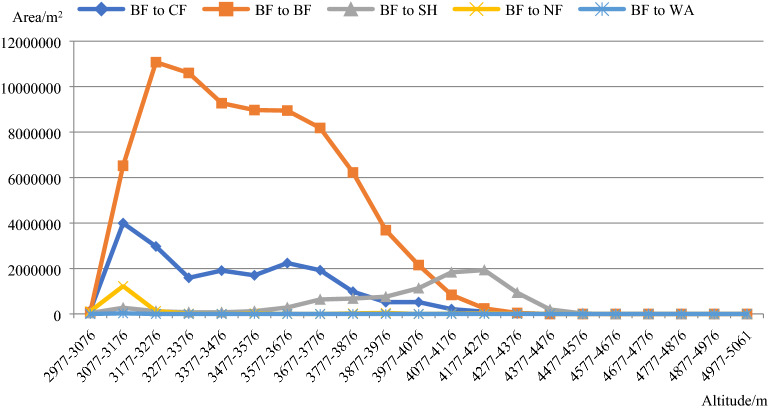
Distribution of the changed and unchanged CF areas in the 21 altitude gradients between 2013 and 2015. The figure is generated using the Microsoft Office 2016 (http://www.office.com).

Figure 6 shows that the transformation of BF into CF was widely distributed in the 3077–4276 m altitude gradient. Above 4277 m, this change rarely occurred. The unchanged BF area was mainly distributed in the 3077–4176 m altitude gradient. The transformation of BF into SH was widely distributed in the 3577–4476 m altitude gradient. The transformation of BF into NF occurred in the 3077–3176 m altitude gradient. Very little BF was transformed into WA in the study area.

**Figure 7:**
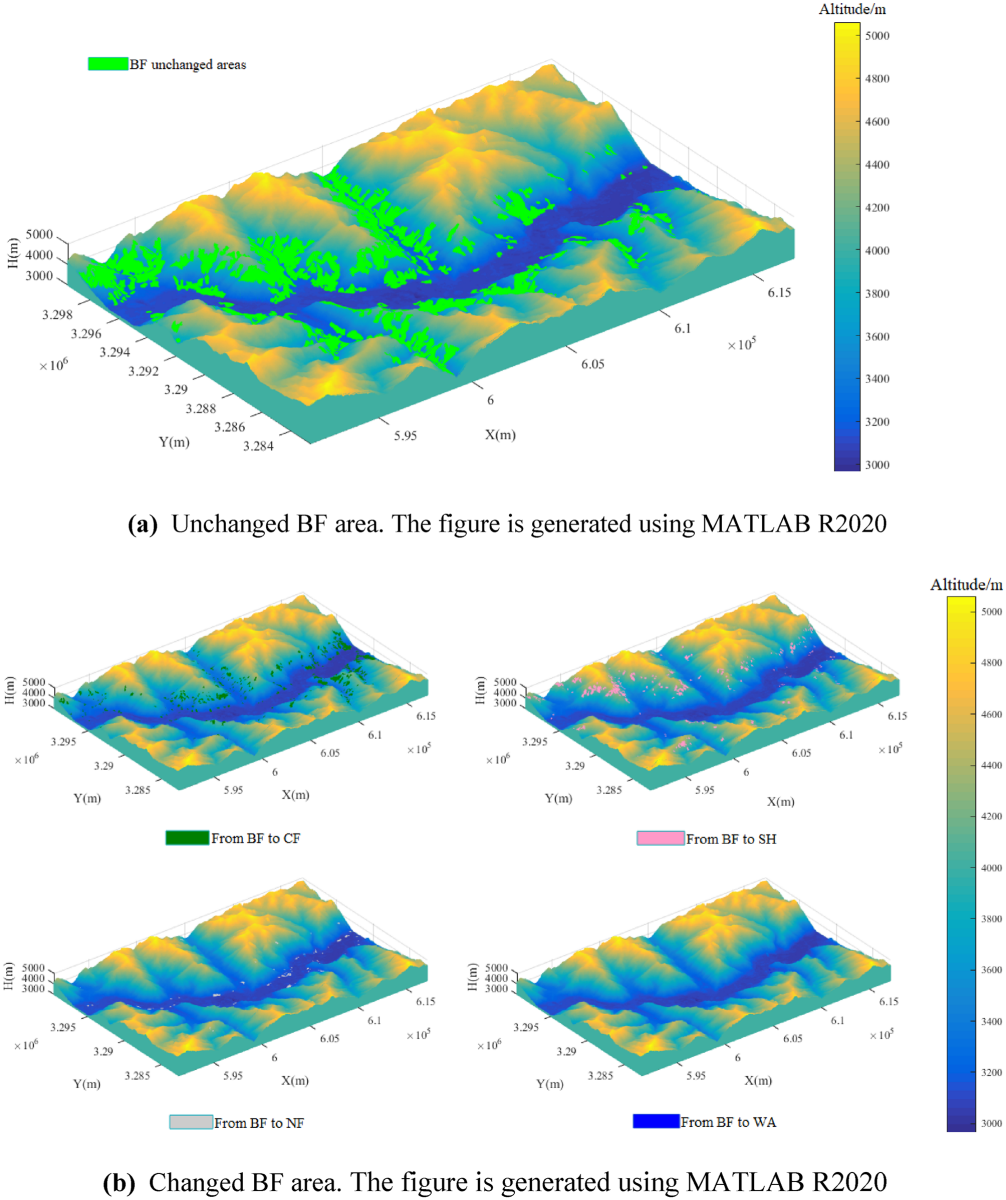
**(a)** Unchanged BF area. The figure is generated using MATLAB R2020 (http://www.mathworks.com). **(b)** Changed BF area. The figure is generated using MATLAB R2020 (http://www.mathworks.com).

Figure 7a shows that the unchanged BF area was mainly distributed in the western part of the study area, and most of the unchanged BF area was located on the eastern, western and northern aspects of the mountains. Very little of the unchanged BF area occurred on the southern aspect of the mountains.

Figure 7b shows that the transformation of BF into CF mainly occurred in the central and eastern parts of the study area, at altitudes of 3000 to 4000 m. The distribution was not directly related to the aspect direction. The transformation of BF into SH mainly occurred in the northwestern part of the study area, and very little occurred in the southern and eastern parts of the study area. Barely any of this transformation occurred on the western and northern aspects of the mountains. The transformation of BF into NF mainly occurred along the riverbank. As with the CF, very little BF was transformed into WA in the study area.

**Figure 8:**
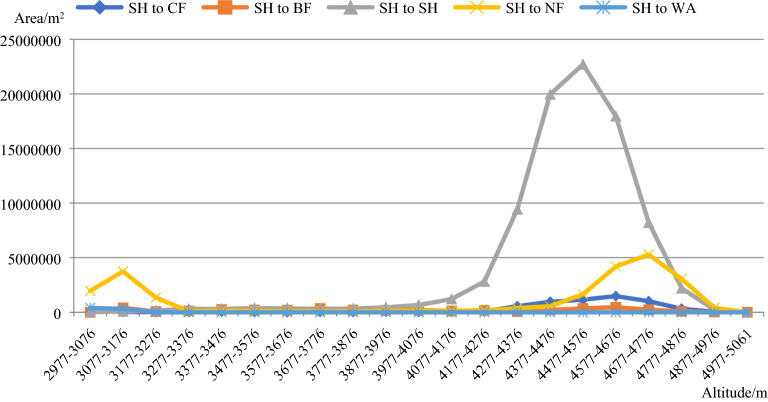
Distribution of the changed and unchanged SH area in the 21 altitude gradients between 2013 and 2015. The figure is generated using the Microsoft Office 2016 (http://www.office.com).

Figure 8 shows that above 4077 m, the SH was transformed into both CF and BF, and there were also SH areas that remain unchanged. In the 2977–3276 m and 4277–4976 m altitude gradients, the SH was transformed into NF, and the SH was only transformed into WA in the 2977–3176 m altitude gradient.

**Figure 9:**
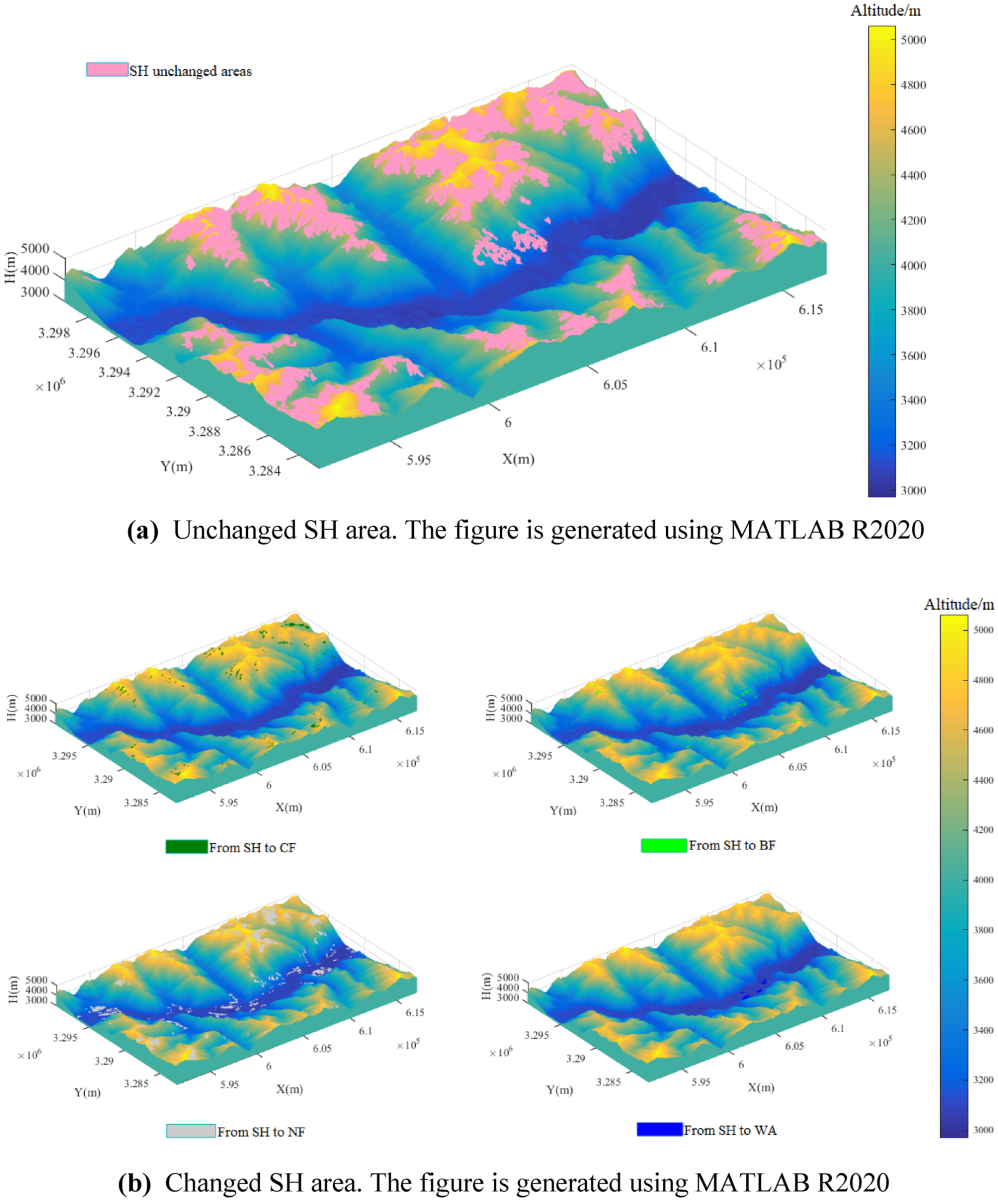
**(a)** Unchanged SH area. The figure is generated using MATLAB R2020 (http://www.mathworks.com). **(b)** Changed SH area. The figure is generated using MATLAB R2020 (http://www.mathworks.com).

Figure 9a shows that the unchanged SH area was mainly distributed in the northern and southwestern parts of the study area, and they mainly occurred on the western and southern aspects of the mountains.

Figure 9b shows that the SH was mainly transformed into CF on the northern and western aspects of the mountains. The SH was widely transformed into CF in the study area, but the transformation was scattered across the study area, and no large areas of transformation occurred. Both at high and low altitudes, the SH was transformed into NF, and mainly occurred on the southern and eastern aspects of the mountains. The SH was transformed into WA where water storage projects were implemented.

## Discussion

In this study, Nyingchi County was selected as the study area. The Landsat OLI images and DEM datasets were used as the main datasets for studying the LULCCs in Nyingchi County. The main goal of this study was to determine the spatial distributions of and changes in the land cover types in the study area. The results provide a better understanding of the dynamic LULCCs that have occurred in recent years. In this study, the land cover change detection was no longer restricted to the planar scale. As the terrain conditions of the TP are quite unique compared to other places on Earth, in order to study the spatial changes in the land cover types in the study area, the use of DEM data was introduced in this study. Twenty LUC areas and five LUU land use areas on curved land surface areas were calculated. The forest land cover change results were optimized, and the dynamic changes in the forest land cover areas were analyzed. Based on the result, the total forest area in the study area in 2015 decreased. In 2015, the CF was mainly transformed into SH and BF, while the BF was the main land cover that transformed into CF. The total CF area decreased by 25.14% compared with the CF areas in 2013. For the SH in the study area, most of the SH was transformed into NF in 2015, and most of the areas of SH increase in 2015 were transformed from CF. The SH area increased by 14.62% in 2015. In 2015, the BF area increased by 12.65%, of which, most of the BF area was transformed into CF, and most of the BF increased were due to the transformation of CF. The area of CF transformed into BF was about twice that of the area of BF transformed into CF.

The highest elevation in the study area is 5061 m. Based on altitude, the study area was divided into 21 altitude gradients. The curved land surface areas of the land cover types in the different altitude gradients and the changed and unchanged areas were calculated. Based on the results of the change detection, the spatial dynamic changes in the land cover types were analyzed. The results indicate that the transformation of BF into CF mainly occurred in the western part of the study area at altitudes of 3077–3976 m. The transformation of CF into SH mainly occurred on the eastern and southern aspects of the northern mountains at altitudes of 3077–3176 m and 3877–4576 m. The transformation of BF into CF mainly occurred in the central and eastern parts of the study area at altitudes of 3077–4276 m. The transformation of SH into NF occurred at altitudes of 2977–3276 m and 4277–4976 m, these transformations are distributed on the southern and eastern aspects of the mountains.

Most of the previously studies on the monitoring the dynamic changes of the forestry land cover type focused on two-dimensional level, which cannot accurately reveal the spatial distribution of vegetation coverage. Due to terrain impacts on the vegetation distribution, for the forest that located at the mountainous region, for study of land use classification and change detection, the spectral information of the remote sensing images was not sufficient. Therefore, the DEM data was used in the study to extraction the spatial distribution and dynamic change of the different land use type of the study area in 2013 and 2015. The results of this study reveal that the proposed method is an effective means of studying the LULC in complicated terrains. These results provide a better understanding of the spatial distributions of and changes in the land cover types, which provides a more intuitive understanding of the dynamic changes in land cover type on the TP. According to the study results, monitoring the mountainous forest on spatio-temporal level and deep mining the three-dimensional information of the forest resource dynamic changes can help formulate accurate forest protection policies and realize the sustainable management of the forest resources. However, there are still several problems regarding the dynamic change in the land cover type that should be studied further. In this study, we focused on a short time series of LULC, and the driving factors of the land use change were not investigated and discussed in this study. Further research on the driving factors and the LULC’s impact on the environment and climate is necessary.

## Conclusions

In this study, the LULCCs from 2013 to 2015 in Nyingchi County on China’s Tibetan Plateau were analyzed. In this study, DEM data were used in the calculations of the changed and unchanged curved land surface areas, and thus, the land cover change results were modified. In the places like the TP, where the terrain is complex, the calculation of the curved land surface areas is close to the real conditions. The altitude of the study area was divided into 21 gradients, and the land cover changes from 2013 to 2015 were analyzed. The method proposed in this study can effectively extract the spatial dynamic changes in the land cover types in the study area. Based on the three-dimensional graphics of the land cover change results, the graphics reveal the land cover change information for the true three-dimensional terrain conditions. The three-dimensional graphics obtained using the method proposed and applied in this study have a rather good visual effect. Because there are no shaded sides, the distributions of both the changed and unchanged areas can be easily observed, and thus, these graphics can be effectively used to analyze the spatial dynamic land cover changes.

## Materials and methods

### Satellite images and data processing

Landsat 8 images were used in this study. The Landsat 8 satellite was launched by the United States Geological Survey (USGS) and the National Aeronautics and Space Administration in 2013, and it contains two sensors: the operational land imager (OLI) and the thermal infrared sensor. The Landsat OLI image contains nine bands with a resolution of 30 m, and a full-color band with a resolution of 15 m. The images used in this study were downloaded from the USGS and the Internal Scientific Data Service Platform of China. Based on the study’s goal of land cover change detection, two sets of images taken in 2013 and 2015, with cloud cover of less than 5%. The Landsat OLI images were obtained during the vegetation growing season. Before the remote sensing image classification and land cover change detection, the images were processed using ENVI 5.3 software. The image processing included radiometric, atmospheric, geometric, and topographic corrections. After the atmospheric correction was made, the vegetation spectrum curve was close to the real vegetation spectrum. Images that cover the Yarlung Zangbo River Basin were obtained.

**Figure 10:**
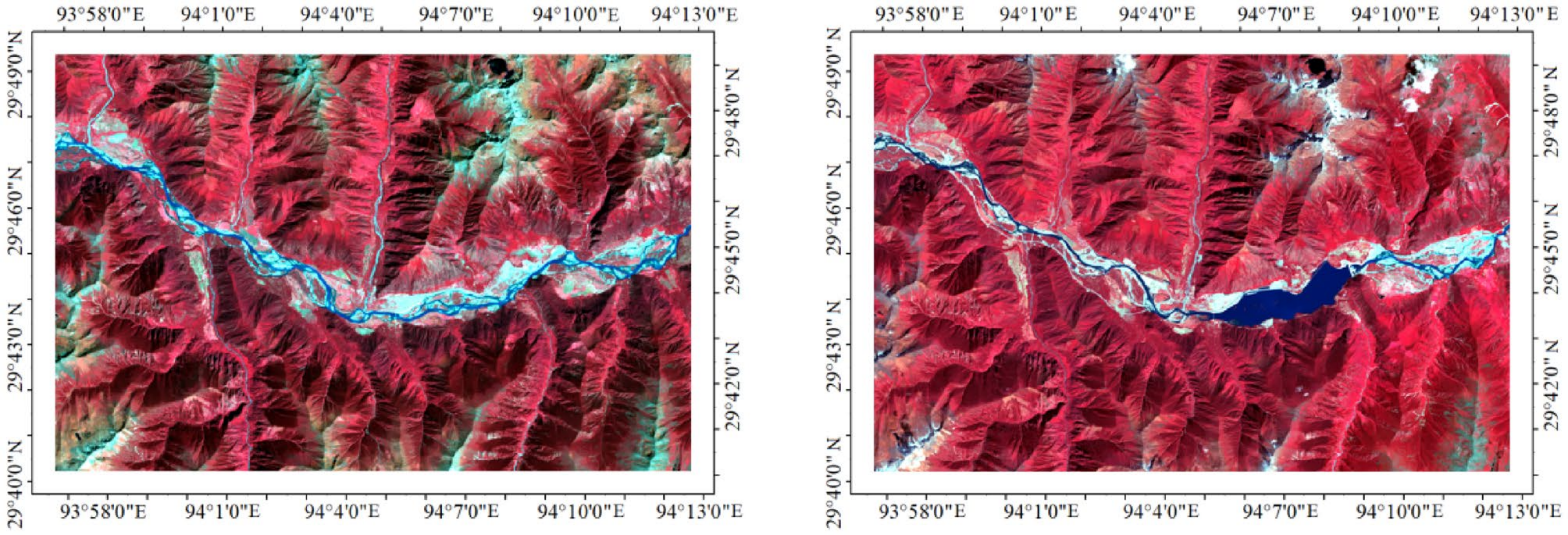
Remote sensing images of the study area. The images can be obtained from the websit of the USGS (http://glovis.usgs.gov/). The figure is generated using ArcGIS 10.3 (http://www.esri.com/software/arcgis/arcgis-for-desktop).

The DEM dataset was also used in this study. The spatial resolution of the DEM was 1 arcsec. In order to make it consistent with the remote sensing images, the DEM dataset was resampled. The resample spatial resolution was 30 m, and cubic convolution interpolation was used for the dataset resampling. However, even though the remote sensing images were geo-referenced, the images and the DEM dataset did not line up exactly. In order to match the different images, the remote sensing images were resampled based on the DEM dataset using the grid snap method, after which all of the images lined up exactly. The remote sensing images covering the study area are shown in Fig. 10.

### Curved surface area calculation

In this study, in order to calculate the curved land surface areas of the LUU and the LUC, the thematic change workflow of ENVI software was used to extract the vector boundaries of the LUU and LUC. The vector boundaries were used to trim the study area’s DEM data, and thus, 20 LUC DEM and 5 LUU DEM were obtained. All DEM data were in tiff. Format; they were processed using Global Mapper and transformed into point cloud data. Then, the curved land surface areas of the 20 LUCs and 5 LUUs were calculated. The format of the point cloud data was defined as:

1 $$ dem = \left[ {\begin{array}{*{20}c} {y_{1} } & {x_{1} } & {h_{1} } \\ {y_{1} } & {x_{2} } & {h_{2} } \\ \vdots & \vdots & \vdots \\ {y_{1} } & {x_{m} } & {h_{m} } \\ {y_{2} } & {x_{1} } & {h_{m + 1} } \\ {y_{2} } & {x_{2} } & {h_{m + 2} } \\ \vdots & \vdots & \vdots \\ {y_{2} } & {x_{m} } & {h_{2m} } \\ \vdots & \vdots & \vdots \\ {y_{n} } & {x_{1} } & {h_{(n - 1)m + 1} } \\ {y_{n} } & {x_{2} } & {h_{(n - 1)m + 2} } \\ \vdots & \vdots & \vdots \\ {y_{n} } & {x_{m} } & {h_{nm} } \\ \end{array} } \right] $$

where *y*_*i*_ (*i* = 1, 2, …, *n*) represents the abscissa values and *x*_*i*_ (*i* = 1, 2, …, *n*) represents the ordinate values of the spatial rectangular coordinate system O-XYZ. *h*_*k*_ (*k* = 1, 2, …, n*m) is the height of the altimetric point, *n* is the altimetric points position on the east-–west line; and *m* is the altimetric points position on the north–south line. The corresponding coordinate values can be calculated using *m* and *n*, which are obtained based on the DEM matrix.

2 $$ y_{1 \times n} = \left[ {\begin{array}{*{20}c} {y_{1} } & {y_{2} } & \cdots & {y_{n} } \\ \end{array} } \right] $$

3 $$ x_{1 \times n} = \left[ {\begin{array}{*{20}c} {x_{1} } & {x_{2} } & \cdots & {x_{m} } \\ \end{array} } \right] $$

The spatial resolution of the DEM is 30 m, which means y_i_ is 30 m larger than y_i−1_, and x_j_ is 30 m larger than x_j−1_. Thus, the complete data grid is defined by the following matrixes:

4 $$ y_{m \times n} = \left[ {\begin{array}{*{20}c} {y_{1} } & {y_{2} } & \cdots & {y_{n} } \\ {y_{1} } & {y_{2} } & \cdots & {y_{n} } \\ \vdots & \vdots & \vdots & \vdots \\ {y_{1} } & {y_{2} } & \ldots & {y_{n} } \\ \end{array} } \right], $$

5 $$ x_{m \times n} = \left[ {\begin{array}{*{20}c} {x_{1} } & {x_{1} } & \cdots & {x_{1} } \\ {x_{2} } & {x_{2} } & \cdots & {x_{2} } \\ \vdots & \vdots & \vdots & \vdots \\ {x_{m} } & {x_{m} } & \cdots & {x_{m} } \\ \end{array} } \right]. $$

Each grid point is related to a height value, and the height value matrix is defined as

6 $$ h_{m \times n} = \left[ {\begin{array}{*{20}c} {h_{11} } & {h_{12} } & \cdots & {h_{1n} } \\ {h_{21} } & {h_{22} } & \cdots & {h_{2n} } \\ \vdots & \vdots & \vdots & \vdots \\ {h_{m1} } & {h_{m2} } & \cdots & {h_{mn} } \\ \end{array} } \right] $$

The data set $\left( {y,\;x,\;h} \right)_{m \times n}$ can be expressed as the function $h = f\left( {y,x} \right)$. In this study, the gradients in both the east–west direction and the south-–north direction were calculated using the gradient function $\left[ {fy,\;fx} \right] = gradient\left( {h,\;step,step} \right)$. The average space in every direction is a step, and because the resolution of the DEM data is 30 m, the step value was set as 30 before the calculation. The element of the curved surface areas can be calculated by formula (Eq. 7). The sum of all the elements is the area of the curved land surface. The curved land surface areas of 20 LUCs and 5 LUUs were used to analyze the dynamic change in the land cover in the study area.

7 $$ ds = \sqrt {1 + \left( {\frac{\partial f}{{\partial y}}} \right)^{2} + \left( {\frac{\partial f}{{\partial x}}} \right)^{2} } dydx = \sqrt {1 + \left( {fy} \right)^{2} + \left( {fx} \right)^{2} } \times step \times step $$
